# Exome sequencing enables diagnosis of X-linked hypohidrotic ectodermal dysplasia in patient with eosinophilic esophagitis and severe atopy

**DOI:** 10.1186/s13223-021-00510-z

**Published:** 2021-01-14

**Authors:** Bhavi P. Modi, Kate L. Del Bel, Susan Lin, Mehul Sharma, Phillip A. Richmond, Clara D. M. van Karnebeek, Edmond S. Chan, Vishal Avinashi, Wingfield E. Rehmus, Catherine M. Biggs, Wyeth W. Wasserman, Stuart E. Turvey

**Affiliations:** 1 Centre for Molecular Medicine and Therapeutics, Dept. of Medical Genetics, BC Children’s Hospital Research Institute, University of British Columbia, Vancouver, BC Canada; 2 BC Children’s Hospital, University of British Columbia, 950 W 28th Ave, Vancouver, BC V5Z 4H4 Canada; 3 Division of Allergy & Immunology, Department of Pediatrics, Faculty of Medicine, University of British Columbia, Vancouver, BC Canada; 4 Department of Pediatrics, Emma Children’s Hospital, Amsterdam University Medical Centres, Amsterdam, The Netherlands; 5 Division of Gastroenterology, Hepatology and Nutrition, Department of Pediatrics, Faculty of Medicine, University of British Columbia, Vancouver, BC Canada; 6 Division of Dermatology, Department of Pediatrics, Faculty of Medicine, University of British Columbia, Vancouver, BC Canada

**Keywords:** X-linked hypohidrotic ectodermal dysplasia, EDA, Atopy, Exome sequencing

## Abstract

X-linked hypohidrotic ectodermal dysplasia (XLHED) is the most common form of ectodermal dysplasia. Clinical and genetic heterogeneity between different ectodermal dysplasia types and evidence of incomplete penetrance and variable expressivity increase the potential for misdiagnosis. We describe a family with X-linked hypohidrotic ectodermal dysplasia (XLHED) presenting with variable expressivity of symptoms between affected siblings. In addition to the classical signs of hypohidrosis, hypotrichosis and hypodontia, the index patient—a 5 year old boy, also presented with a severe atopy phenotype that was not observed in the other two affected brothers. Exome sequencing in the index and the mother identified a pathogenic nonsense variant in *EDA* (NM_001399.4: c.766 C>T; p. Gln256Ter). This study highlights how exome sequencing was crucial in establishing a precise molecular diagnosis of XLHED by enabling us to rule out other differential diagnoses including NEMO deficiency syndrome, that was initially presented as a clinical diagnosis to the family.

## Introduction

**Fig. 1:**
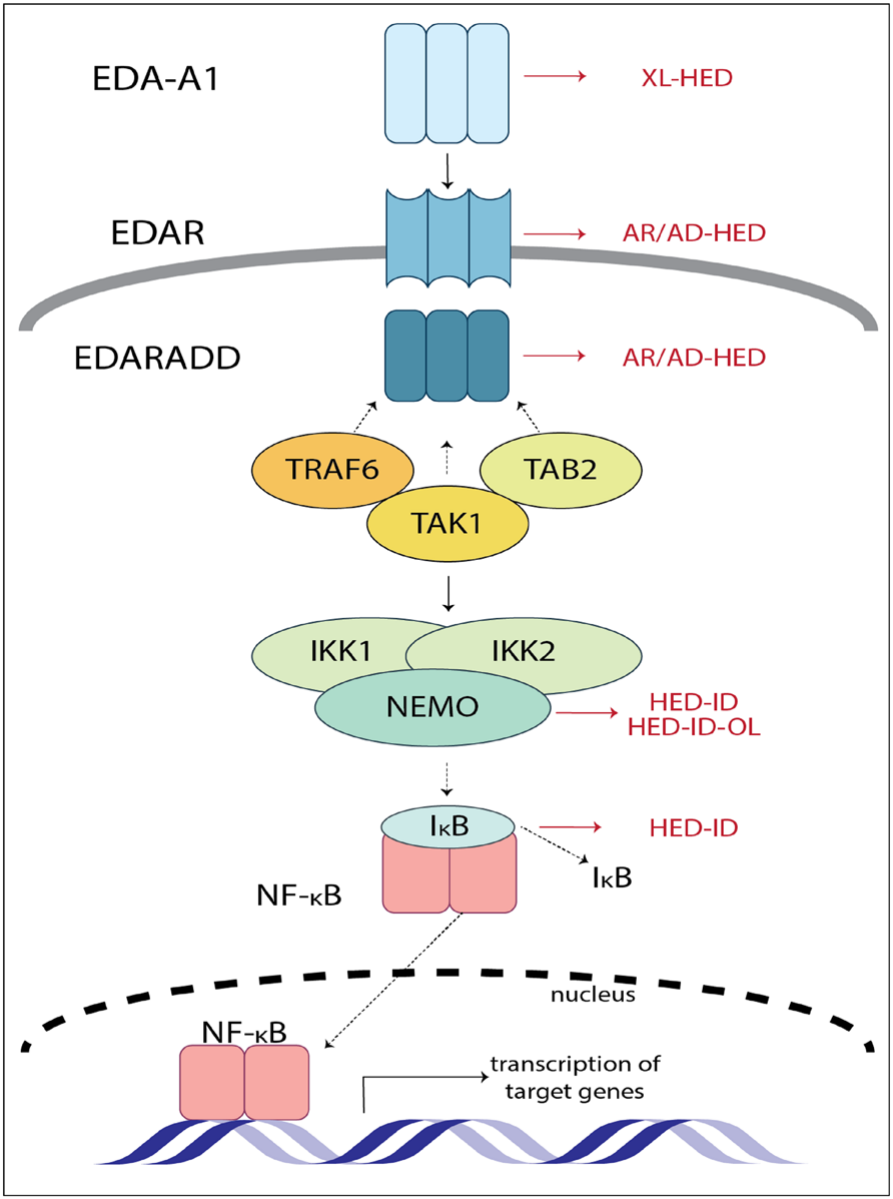
EDA signaling pathway. The trimeric EDA ligand binds to the trimeric EDAR receptor and recruits the adaptor EDARADD, which then forms a complex with TRAF6, TAB2 and TAK1. This results in activation of the IKK complex via TAK1. The activated IKK complex (NEMO, IKK1 and IKK2) ubiquitinates IκB, thereby releasing NF-κB, which can translocates into the nucleus to activate transcription of target genes. The pathway represents protein names and symbols. The HGNC gene symbols are as follows: *EDA* (EDA-A1), *EDAR* (EDAR), *EDARADD* (EDARADD), *TRAF6* (TRAF6), *MAP3K7* (TAK1), *TAB2* (TAB2), *CHUK* (IKK1), *IKBKB* (IKK2), *IKBKG* (NEMO), *NFKB1A* (IκB), *NFKB1* (NF-κB). The different ED types caused by variants in different genes in the EDA signaling pathway are described in red text on the right. Figure [14] adapted from Sadier et al.

Ectodermal dysplasias (EDs) are a group of clinically and genetically heterogeneous disorders characterized by abnormal development of two or more ectodermal structures including teeth, hair, skin, nails, sebaceous glands and other eccrine glands [1]. Amongst a collective group of more than 200 different types of EDs, the hypohidrotic form of ED (HED) is the most common. HEDs are caused by mutations in one of several genes (i.e. genetic heterogeneity) encoding components of the ectodysplasin A (*EDA*) signaling pathway crucial in embryonic ectodermal development (Fig. 1).

HED typically follows one of three possible inheritance patterns: the X-linked form is caused by variants in *EDA* (MIM: 300451) encoding the ligand ectodysplasin A-A1; the autosomal recessive and autosomal dominant forms are caused by variants in the EDA receptor encoded by *EDAR* (MIM: 604095) and in the EDAR-associated death domain which is encoded by *EDARADD* (MIM: 606603) (Fig. 1). Of these, X-linked hypohidrotic ectodermal dysplasia (XLHED) is the most common phenotype occurring in one per 17,000 live births and is distinguished from other ED types by a triad of classical signs—hypohidrosis (reduced ability to sweat), hypotrichosis (sparse thinning hair) and pointed teeth or lack of several teeth (hypodontia or anodontia). While affected hemizygous males are most severely impacted showing the classical trio of signs, heterozygous female carriers display variable expressivity without symptoms or only moderately affected features such as uneven distribution of sweating and defective dentition [1, 2].

Without molecular tests, the clinical and genetic heterogeneity associated with EDs makes diagnosis and management challenging. Incomplete penetrance and variable expression, sometimes even between members of the same family, illustrate the lack of clear genotype–phenotype relationships [3, 4]. Differential diagnoses often include disorders caused by variants in genes belonging to the same pathway presenting with overlapping clinical symptoms. Most notable in this regard are genes downstream of EDA signaling in the TNF-α pathway that lead to NF-κB activation. Variants in *IKBKG* (MIM: 300248; also known as *NEMO*) lead to syndromic features with symptoms of incontinentia pigmenti or classical HED accompanied by immunodeficiency (HED-ID) and/ or osteopetrosis and lymphedema (HED-ID-OL). Because of the associated clinical heterogeneity and variable expression, it is not possible to phenotypically distinguish these disorders from classical HED with certainty [5]. Besides, lack of a genetic diagnosis means the opportunity for appropriate genetic counseling and risk assessment in family members is missed.

## Case report

**Fig. 2:**
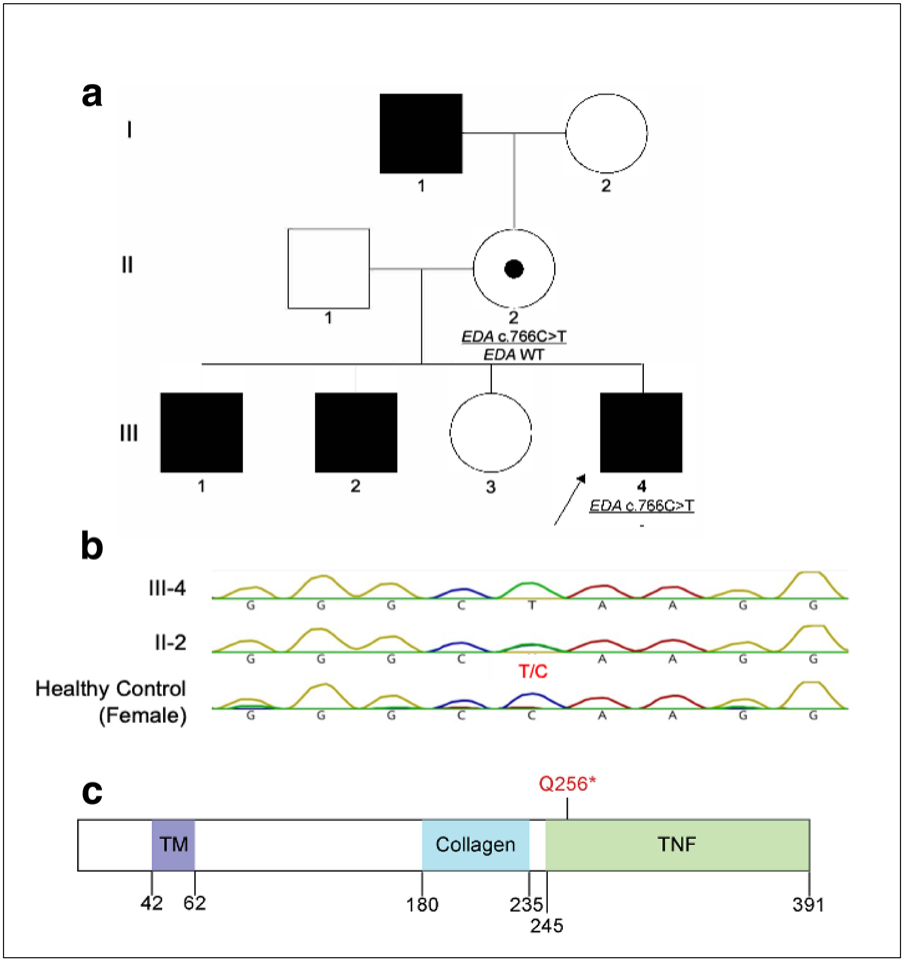
Family pedigree and the *EDA* c.766C > T (p.Q256*) variant. **a** Family pedigree. **b** Sanger sequencing of whole blood from the male index patient (III-4), the mother (II-2) and a healthy female control showing that the index (III-4) is hemizygous for the *EDA* c.766C > T variant whereas the mother (II-2) is heterozygous. The female healthy control shown here is homozygous for the reference allele. **c** Representative structure of the EDA protein (full-length EDA-A1 isoform with 391 amino acids) showing the position of the p.Q256* variant within the TNF domain

We report the application of Whole Exome Sequencing (WES) for the ultimate diagnosis of XLHED in a family due to a pathogenic nonsense *EDA* variant (Fig. 2a). The index is a 5-year-old male (III-4) presenting with classical HED signs including sparse thin hair, hypodontia and/ or pointed teeth and overheating with an inability to sweat. In addition, the index is affected by severe atopy with eosinophilic esophagitis. His atopic diathesis is characterized by multiple IgE-mediated food allergies, asthma, allergic rhinoconjunctivitis and severe atopic dermatitis. Additional details about the atopy features in the index and their management are presented in Table 1. The mother (II-2) has a milder HED phenotype (without atopy) presenting with pointed teeth and mild overheating with somewhat reduced sweating. The index's two male siblings (III-1, III-2) show similar HED symptoms (without any atopy) while a female sibling (III-3) is unaffected. The index’s maternal grandfather (I-1) is also reported to be affected.

**Table 1:** Clinical features of atopy in the index: The atopic conditions presented in the index patient (III-4) and notes on their management are described

Condition	Manifestation	Management
Atopic dermatitis	Onset in the first few months of life, challenging to manage with multiple flares over the years	Chronic use of varying potency topical steroids combined with oral antibiotics for recurrent episodes of group A streptococcal impetigo
Eosinophilic esophagitis	Diagnosed at 3yo based on difficulty tolerating solid food and repeated vomiting combined with multiple upper GI biopsies repeatedly showing active esophagitis with increased intraepithelial eosinophils	Avoidance of foods with documented IgE sensitization (cow's milk, egg, peanut, and peas), use of an elemental formula, and ultimately the need for oral viscous budesonide combined with a proton pump inhibitor
Asthma	Diagnosed at 3yo requiring 3 hospital admissions, multiple emergency room visits and repeated courses of oral corticosteroids	Responsive to a maintenance controller combination of regular inhaled corticosteroids and montelukast with inhaled salbutamol used occasionally as a reliever. Recommended avoidance of environmental allergens where IgE sensitization was documented (tree, grass, and weed pollen, mold, cat, dog, and dust mite)
Food allergy	Diagnosed at 1yo on the basis of a history of anaphylactic reactions following exposure combined with positive epicutaneous testing to cow's milk, egg, peanut, and peas	Strict avoidance and ensuring availability of an epinephrine autoinjector. Attempted food challenges were difficult to interpret due to the severity of his skin inflammation
Allergic rhinoconjunctivitis	Classic symptoms and signs combined with positive epicutaneous testing to tree, grass, and weed pollen, mold, cat, dog, and dust mite	Recommended avoidance of environmental allergens where IgE sensitization was documented (tree, grass, and weed pollen, mold, cat, dog, and dust mite). Nasal corticosteroid sprays and oral non-sedating antihistamine

A possible clinical diagnosis of NEMO deficiency ED initially triggered referral of the index patient to clinical immunology because of his history of infections; including recurrent Group A streptococcal skin infections requiring multiple courses of antibiotics and episodes of oral candidiasis. Although it is important to emphasize that increased susceptibility to pyogenic bacteria, viruses and nonpathogenic mycobacterial infections are the classic features of NEMO deficiency ED [6]. However, WES in the index and the mother identified a previously known pathogenic nonsense variant (Clinvar Accession: VCV000177947.1) in the *EDA* gene [NM_001399.4: c.766 C > T; NP_001390: p. Gln256Ter; rs727504417]. The index is hemizygous for the variant and the mother is heterozygous for the variant. Subsequent Sanger sequencing confirmed these findings (Fig. 2b). The variant results in a premature translational stop signal at amino acid position 256 in the TNF (Tumor Necrosis Factor) homology domain and is predicted to result in an absent or truncated protein product (Fig. 2c). The C-terminal TNF homology domain is the receptor binding domain of the protein and mutations in this domain are known to impair binding of both EDA protein isoforms (EDA-A1 and EDA-A2) to their receptors. Identification of the pathogenic *EDA* nonsense variant had a number of clinical benefits for the family including ending their search for a diagnosis, advice on avoiding complications of hyperthermia, informing future specialty assessments, reproductive counseling, and reassurance that prognosis for normal growth, development, and lifespan is excellent.

## Discussion

The presence of atopic symptoms in the index patient raised the question of whether these additional clinical features are a phenotypic expansion underlying the variable expressivity associated with EDs or whether they indicate an additional differential diagnosis in the index. In order to rule out the possibility of any competing or secondary diagnoses in the index patient, we specifically looked for damaging variants in genes that could lead to NEMO deficiency ED (*IKBKG*) or primary atopic disorders with significant skin involvement and multiple allergies such as those caused by damaging variants in *FLG, DSP, DSG1 and SPINK5*, but did not identify any clinically relevant variants [7]. While a secondary diagnosis cannot be completely ruled out considering the limitations of WES and the possibility of variants in non-coding regions or genes associated with primary atopic disorders that are currently unknown, we believe that the severe atopy seen in the index patient is likely secondary to his ectodermal dysplasia.

Two different studies performing retrospective analysis of individuals with hypohidrotic/anhidrotic ectodermal dysplasias have reported that these individuals have a significantly increased prevalence of atopic disorders compared to the general population [8, 9]. The study by Guazzarotti et al*.* investigated the phenotypic spectrum in 45 Italian male subjects with molecularly confirmed XLHED and found that 71.1% showed at least one allergic manifestation. However, only 3% of the patients had food allergy and none had eosinophilic esophagitis [8]. Similarly, a retrospective survey-based study of 347 families who were members of the National Foundation for Ectodermal Dysplasias found greater reported prevalence of symptoms suggestive of atopic disorders among children with ED syndromes than the general pediatric population [9]. Several other case reports of HED patients presenting with signs of allergic disease also support these findings [10, 11]. Though the specific mechanism(s) underlying the increased susceptibility and development of allergic disease in ectodermal dysplasias is unclear, disruption and dysfunction of the skin barrier are generally considered to contribute to the pathogenesis [7].

## Conclusion

In conclusion, we report a case of XLHED that presented with classical HED signs of hypohidrosis, hypotrichosis and hypodontia as well as severe atopy. These findings emphasize the variable expressivity of XLHED between affected individuals, in this case within the same family. They highlight the need for in-depth evaluation and reporting of clinical features to expand our understanding of the phenotypic spectrum associated with XLHED. We would like to draw attention of health care practitioners and researchers in allergy, asthma and clinical immunology to the importance of applying unbiased genetic testing modalities like whole exome sequencing towards the diagnosis of patients that present with vast phenotypic heterogeneity or unusual severity of certain symptoms. By presenting an accurate molecular diagnosis to the family, we can now provide them with an answer to better understand their medical condition as well as its genetic implications—steps toward a personalized medicine approach with accurate genetic counselling and tailored clinical management.

## Methods

### Subjects

The family was enrolled into the GARD (Genetic Alterations in Rare Diseases) research study (UBC IRB approval H09-01,228). Informed written consent was obtained from the index patient and the mother for their participation in the study, sample collection, whole exome sequencing, data analysis and publication of findings.

### Sequencing and genomic analysis

Genomic DNA was isolated from peripheral blood using standard protocols and exome sequencing for the mother and the index patient was performed on an Illumina platform. Exome Sequencing data was analyzed using an updated version of our in-house, open-source, semi-automated bioinformatics pipeline that has been previously described. [12, 13] Once a candidate variant list was generated by the pipeline, variants identified through the X-linked inheritance models were first assessed as the family pedigree was strongly suggestive of an X-linked inheritance pattern. Next, the candidate variant list was screened for variants in *IKBKG* (to rule out NEMO deficiency) as well as primary atopic disorder genes. In addition, a phenotype-driven approach was also employed to identify variants in genes that may be associated with clinical features such as eczema, allergy, asthma, dermatitis, atopy (associated MeSH and HPO terms) present in the index patient.

Confirmation of the *EDA* variant identified through exome sequencing in the index patient and the mother was done using Sanger sequencing.

### Primer sequences

F: 5′ TCCCTTGCTACAGCTGTGTG 3′

R: 5′ CGTATGCCAACGGTACCTCA 3′

## Data Availability

Not applicable.

## Abbreviations

DSG1: Desmoglein 1
DSP: Desmoplakin
ED: Ectodermal dysplasia
EDA: Ectodysplasin A
EDAR: EDA receptor
EDARADD: EDAR-associated death domain
ES: Exome sequencing
FLG: Filaggrin
IKBKG: Inhibitor of nuclear factor kappa B kinase regulatory subunit gamma
NEMO: NF-κB essential modulator
NF-κB: Nuclear factor kappa B
SPINK5: Serine peptidase inhibitor Kazal Type 5
TNF: Tumor necrosis factor
XLHED: X-linked hypohidrotic ectodermal dysplasia
